# Do restaurants comply with reduced salt requests from consumers ordering on meal delivery apps?

**DOI:** 10.1186/s12889-023-16939-3

**Published:** 2023-10-13

**Authors:** Chao Song, Wenyue Li, Ying Cui, Beisi Li, Zhongdan Chen, Paige Snider, Ying Long, Ailing Liu, Gauden Galea

**Affiliations:** 1 National Institute for Nutrition and Health, Chinese Center for Disease Control and Prevention, Beijing, 100050 China; 2 School of Architecture, Tsinghua University, Beijing, China; 3 World Health Organization Representative Office in China, Beijing, China

**Keywords:** Salt reduction, Sodium, Restaurants, Consumer demand, Meal delivery app

## Abstract

**Background:**

Chinese urban residents consume more salt from meals prepared outside home than in the past. The purpose of this study is to understand Chinese consumer demand for salt reduction as expressed through their orders on meal delivery apps (MDAs), restaurants’ willingness to promote salt reduction, and the extent to which restaurants comply with reduced salt requests.

**Methods:**

We analyzed consumer comments extracted from 718 restaurants on a Chinese MDA called ELEME for orders made in the July-December 2020 timeframe. A self-designed questionnaire was distributed to the restaurant managers to assess restaurants’ attitude towards salt reduction upon signing up for the study, and laboratory validation was conducted to test whether dishes ordered with reduced salt requests by consumers actually contained less salt.

**Results:**

A total of 25,982 (0.7%) orders out of 3,630,798 orders contained consumer comments. Of the consumer comments, 40.6% (10,549) were about requests for less salt in dishes. Totally 91.5% of 421 surveyed restaurants showed a willingness to respond to consumers’ reduced salt requests. The median sodium content measured in the reduced-salt dishes by the laboratory was significantly lower than that in their regular salt counterparts (*P* < 0.05).

**Conclusions:**

We observed substantial consumer demand for salt reduction while ordering meals on the MDA and that restaurants did, in response, reduce the sodium content in the meals they provided. As meals delivered via MDAs comprise an increasing proportion of outside foods consumed, there is an opportunity for public health experts and policy makers to work with MDAs and restaurants to promote healthier food selections.

**Trial registration:**

ChiCTR2100047729.

## Background

Excess sodium intake is associated with increased risks of hypertension, which is a risk factor for many non-communicable diseases, such as cardiovascular diseases (CVDs), stroke, and kidney diseases [1–6]. Data from the World Health Organization (WHO) estimates that 1.13 billion people worldwide suffer from hypertension [2]. A recent national survey in China showed that the prevalence of hypertension in Chinese adults was 27.5%, an increase of 2.3% from 2012 to 2018 [7]. CVD remains the top cause of death in China, with the annual number of CVD-related deaths increasing from 2.51 million to 3.97 million between 1990 and 2016 [8]. Evidence shows that reducing sodium intake can significantly reduce blood pressure in adults with or without hypertension, which can reduce the risk of CVD and stroke [9, 10]. The WHO has recommended a 30% relative reduction in mean population salt intake by 2025, and in the Healthy China 2030 Planning Outline and the National Nutrition Plan 2017–2030, China has set a target of a 20% reduction in mean population salt intake by 2030 [11, 12].

The primary dietary source of sodium is salt. Historically, most sodium consumption by people in China was from salt added during food preparation. The 2015 China National Chronic Noncommunicable Disease and Nutrition Surveillance Report showed that adults in China (> 18 years) on average consumed 9.3 g of salt per day from home cooking alone, exceeding the 5 g per day recommended by the WHO, the Chinese Dietary Guideline, and the Healthy China Action Plan 2019–2030 [1, 7, 13, 14]. Previous studies showed that the amount of sodium consumed from processed foods has been growing, and foods prepared outside the home had more sodium than that from home-made foods [15, 16]. Trends indicate that eating out has become increasingly popular among Chinese urban residents [17]. A recent national survey showed that for people aged 6 years or older, nearly half (46.3%) had at least one meal prepared outside the home in a typical week; 22.3% of respondents had 9 or more meals per week that were prepared outside the home [7]. Therefore, in contrast to some European countries and the United States that have focused on processed foods to achieve salt reduction targets, the salt reduction strategies taken in China can be more tailored to address salt added during home cooking and meals prepared outside the home [18, 19].

In the past few years, online meal delivery has increasingly become the dominant source of food prepared outside the home, and this trend stayed consistent during the pandemic [20, 21]. However, the available meal delivery apps (MDAs) in the Chinese market usually provide little to no nutritional information to guide consumers towards healthier options. If consumers wish to express a dietary preference (e.g., less salt), consumers can type a message in a comment box before placing the order. To our knowledge, no existing studies have explored consumer demand for salt reduction for meals ordered on MDAs in a real-world setting, as well as whether and to what extent restaurants would prepare meals according to consumers’ reduced salt requests. Thus, in this study, we attempted to test three research questions: (1) to explore consumers’ dietary preference for salt reduction by analyzing reduced salt requests made by consumers through the MDA; (2) to assess restaurants’ willingness to provide reduced-salt dishes through a survey with restaurant managers; (3) to test restaurants’ actions to reduce salt content in dishes if requested by consumers. The findings from our study provided insights of restaurants’ response to reduced salt requests made by consumers when ordering meals on MDAs. Given the rapid change of food environment in China and Chinese consumers’ increased reliance on MDAs for daily meals, these results can also contribute to the ongoing salt reduction efforts at the population-level across the country, as well as the rest of the world where are undergoing similar digitalization.

## Methods

### Study Design

The current study was a part of our whole project in which we tested the effectiveness of changes to the choice architecture in increasing consumers’ uptake of reduced-salt dishes on ELEME, one of the largest MDA platforms in China [20]. The overall research involved three treatments that included a conventional educational health message of “no more than 5 g of salt per day” on the top of the ordering page, a salt submenu for individual dishes for consumers to indicate a choice between “reduced salt” and “regular salt”, and changes to the default setting of either “reduced salt” or “regular salt”. Participating restaurants were assigned to different combinations of the three treatments to create five intervention groups: (1) with health message; (2) salt submenu with default on “regular salt”; (3) salt submenu with default on “reduced salt”; (4) salt submenu with default on “regular salt” combined with a health message; and (5) salt submenu with default on “reduced salt” combined with a health message.

### Data sources

We collected data from three sources to support the current analysis. These data sources were as follows: (1) consumer comments made on the MDA for the participating restaurants on the MDA for the 6-month period prior to the study intervention; (2) an online survey with restaurant managers that assessed their attitude and willingness to support salt reduction interventions; and (3) laboratory testing of sodium content in samples of “regular salt” and “reduced salt” versions of dishes randomly collected from participating restaurants.

### Restaurant recruitment

We used two methods to recruit restaurants on a voluntary basis. Restaurants could join the study by responding to an open invitation sent by the MDA to restaurants in seven cities, or by responding to the targeted invitation made through research teams’ existing networks. In total, the 2430 restaurants that signed up were from cities across different geographic regions in China, including Beijing, Changsha, Guangzhou, Hangzhou, Xi’an, Shenyang, Taizhou, Chengdu and Taiyuan, as well as from a restaurant chain with branches across tier-1 and tier-2 cities in China. After signing up, restaurants were encouraged and instructed on how to set up their interface on the MDA as per their assigned intervention group. Those assigned to the salt submenu intervention groups selected individual dishes to be included during the setup. A total of 285 restaurants, or 11.7% of restaurants, correctly completed the setup for the intervention.

To monitor and maintain restaurant compliance, we conducted a weekly check-up of the interface setup for the restaurants per the assigned intervention group. We also created online groups for owners and managers of the restaurants to join through targeted invitations for routine follow-up. In addition, we developed a series of free materials to support salt reduction in the restaurant setting, including questions and answers, handouts, and self-directed training videos, which were distributed through the MDA to all participating restaurants.

### Consumer comments

Of the 2430 restaurants that initially signed up, 718 restaurants (mainly from the cities of Beijing, Taizhou, Shenyang, and Chengdu, along with a restaurant chain) provided operational data to the study team. From these restaurants, we collected consumer comments made when ordering meals on the MDA between July and December 2020, before the start of the intervention in 2021. Two researchers independently extracted and then cross-checked the comments, identifying keywords or phrases (“less salt”, “less sauce”, “not too salty”, and “lighter taste”) related to reducing the amount of salt contained in the food orders for the analysis of consumer demand for salt reduction.

### Online survey with restaurant managers

We distributed a survey questionnaire developed by the research team through the MDA to the restaurant managers upon signing up for the study. During follow-up, we collected 201 questionnaires from restaurants responding to the open invitation, and 220 questionnaires from restaurants responding to the targeted invitation. In the survey, we included questions covering the general restaurant information; knowledge, attitude, and practice related to salt reduction; and the potential impact of salt reduction practices on their businesses. Through the survey results, we assessed restaurants’ willingness to provide reduced-salt dishes.

### Sodium Content Measurement

The sodium content in dishes randomly ordered on the MDA was measured by a qualified testing company using the method documented in the Food Safety National Standard – Determination of Potassium and Sodium in Food (GB5009.91-2017) [22]. The samples were injected into atomic absorption spectrometer after digestion. After flame atomization, sodium absorbed the 589.0 nm resonance line. Within a certain range of concentration, the absorption value was proportional to the sodium content. To conduct the measurement, we developed a sampling plan for the collection of dishes from the 285 restaurants with an interface set up on the MDA (as shown in Table 1). We assumed a standard deviation of 1 g/100 g for sodium content in dishes and expected to achieve 80% power (with two-sided alpha = 0.05) and to detect a change of 0.5 g/100 g in the regular and reduced salt version of a dish [16]. With the assumption of 5 g of sodium per 100 g regular salt dish (5 g/100 g) [23], and the correlation coefficient of 0.1 for sodium content in regular and reduced-salt dishes, the minimum sample size of each layer (per intervention group) was estimated to be 60 – this meant a total sample size of 300 dishes in regular and reduced-salt versions.

A sufficient number of restaurants (more than 60) were included in the intervention groups of “salt submenu with default on “regular salt””, and “with health message”. From those two groups, 60 restaurants were randomly selected for dish sampling, where one dish from each selected restaurant was randomly sampled for sodium content measurement. Due to the small group size, all restaurants in the other three intervention groups, had dishes sampled for sodium content measurement. Each of the restaurants had one to four dishes which were randomly selected until the sampling ceiling of 60 dishes per group was reached. In all cases, different versions of the same dish (regular and reduced salt) were ordered separately but from the same restaurant in order to have comparator dishes.

**Table 1:** The sampling plan for sodium content measurement

Intervention groups	Number of restaurants	Number of dishes with reduced salt option*	Dish sampling	
			Number of restaurants to be sampled	Number of reduced-salt dishes to be sampled
Salt submenu with default on “regular salt”	121	506	60	60
Salt submenu with default on “reduced salt”	32	115	32	60
Salt submenu with default on “regular salt” + Health message	18	100	18	60
Salt submenu with default on “reduced salt” + Health message	34	228	34	60
With health message	80	350	60	60
Total	285	1299	204	300

* To calculate the number of dishes with reduced salt options, for restaurants in the intervention groups with a salt submenu, all dishes with such a submenu setup were included. For restaurants in the intervention group with a health message only and no salt submenu, the number of dishes with a reduced salt option was calculated as the median of the number of dishes in intervention groups with salt submenu, and salt reduction requests were made through consumer comments

### Statistical analysis

The statistical software package SAS version 9.4 was used for data analysis. N (%) was used to present for categorical variables, the *P* value indicated two-sided probabilities, and *P* < 0.05 was considered significant. A Shapiro–Wilk test was used to test the normality of the data distribution, and skewed sodium content data were presented as the median (P25, P75). A Wilcoxon signed-rank test was used to compare the matched pairs design data. The sodium content between the two groups was compared using the Wilcoxon rank sum test, while the Kruskal-Wallis test was used for multigroup data comparison, with the Dwass-Steel-Critchlow-Fligner method applied for further comparisons.

#### Definitions

The restaurants were also categorized based on the primary type of dishes they were serving, including Chinese cuisine, snacks, and others. The provided dishes were categorized into meat dishes, vegetarian dishes, and snacks/staples.

## Results

### Consumer demand for salt reduction

From July to December 2020, a total of 25 982 (0.7%) orders out of 3 630 798 orders contained specific consumer comments. Among these comments, 10 549 (40.6%) were related to salt reduction requests according to specific keywords or phrases such as “less salt/sauce/pickles”, “lighter taste”, “not too much salt”, “no pickles/salt/soy sauce/salty fish”, “less salt appropriately”, and “least salt possible”.

### Restaurants’ willingness to provide reduced-salt dishes

Among the 2430 participating restaurants, 421 (17.3%) restaurants responded to the online survey, 411 (97.6%) expressed a willingness to participate in salt reduction efforts, 406 (96.4%) expressed their willingness to reduce salt in dishes, and 402 (95.5%) expressed their willingness to proactively offer reduced-salt dishes or reduced salt options on the menu (as described in Table 2). Restaurants’ willingness to offer reduced-salt dishes was primarily linked to their awareness of the health benefits of salt reduction (80.5%), followed by the potential to improve their restaurant’s image (37.8%), consumer demand for less salt in dishes (36.3%), and a government requirement or promotion (31.8%). Over half of the restaurant managers held a positive attitude about the long-term impact of salt reduction on their restaurant, and 91.5% of restaurants indicated that they would actively reduce the amount of salt in the dishes if consumers made such a request.

**Table 2:** Restaurants’ feedback on salt reduction

Items	Restaurants responded to the online survey (n, %)
Restaurants responded to the online survey	421 (100.0%)
Restaurants’ feedback on salt reduction	
Willing to participate in salt reduction project	411 (97.6%)
Willing to reduce salt in dishes	406 (96.4%)
Willing to proactively offer reduced salt dishes or reduced salt options on the menu	402 (95.5%)
Willing to reduce salt in dishes if consumers requested	385(91.5%)
Considerations to offer reduced salt dishes	
Consumers’ demand for less salt in dishes	153(36.3%)
Possibility to lower the cost	29(6.9%)
Potential improvement of restaurant image	159(37.8%)
Government requirement or promotion	134(31.8%)
Health benefits from salt reduction	339(80.5%)
Others	13(3.1%)
Attitude about long-term impact to restaurant	
Positive	236(56.1%)
Negative	78(18.5%)
Not sure	107(25.4%)

### Sodium content in dish samples

In total, we sampled 576 dishes from 179 restaurants, among which 297 (51.6%) dishes were ordered with regular salt, and the remaining 279 (48.4%) orders were placed for the same dish but with a request for less salt, to match with their regular salt counterparts accordingly. After eliminating 18 dishes (only one version of a dish, either regular version or reduced-salt version), the rest 558 samples were matched one by one, which meant there were 279 regular salt dishes and 279 reduced-salt dishes.

Table 3 shows the median sodium content in the 279 matched samples. The median sodium content of the 558 samples was 417.5 mg/100 g, and the median sodium content of dishes ordered as regular salt (484.0 mg/100 g) was higher than that (352.0 mg/100 g) of dishes ordered as reduced salt (*P* < 0.05). There was a significant difference in the median sodium content between the regular salt and reduced salt versions of dishes across each subgroup (*P* < 0.05). No significant difference was observed in the median sodium content of the reduced salt dishes ordered by the subgroup of the intervention group, the subgroup of restaurants (with or without the salt submenu setup), or the subgroup of the dish category (*P* > 0.05). When we compared results across restaurant categories, the sodium content of dish samples ordered from Chinese cuisine restaurants was generally higher than that ordered from restaurants specializing in other types of cuisine (*P* < 0.05), regardless of whether the dishes were ordered as regular salt or reduced salt.

The overall difference in median sodium content between the 279 matched dish samples was 90.0 mg/100 g. However, there was no significant absolute difference in sodium content between dishes ordered as regular and reduced salt when compared within the subgroup of the intervention group, the subgroup of restaurants with or without the salt submenu setup, the subgroup of restaurant category, or the subgroup of dish category (*P* > 0.05).

**Table 3:** The sodium content of the matched dish samples (mg/100 g)

Subgroups		Number of matched dishes	Median of sodium content (P25, P75)	Median of sodium content of dishes ordered as reduced salt (P25, P75)	Median of sodium content of dishes ordered as regular salt (P25, P75)	*P*	Absolute difference of sodium content (P25, P75)
Intervention group	Salt submenu with default on “regular salt”	54	359.5(299.0,496.5)	315.0(269.0,422.0)	399.0(334.0,578.0)	< 0.0001	72.0(38.0,123·0)
	Salt submenu with default on “reduced salt”	54	440.0(317.0,572.0)	376.0(249.0,490.0)	513.5(361.0,626.0)	< 0.0001	94.5(52.0,234.5)
	Salt submenu with default on “regular salt” + Health message	61	422.0(321.0,581.0)	338.0(231.0,466.0)	484.0(379.0,662.0)	< 0.0001	108.0(45.8,184.0)
	Salt submenu with default on “reduced salt” + Health message	58	445.0(338.0,619.0)	356.0(281.0,493.0)	498.0(392.0,660.0)	< 0.0001	93.0(37.0,170.0)
	With health message	52	417.0(285.0,645.0)	376.0(258.0,510.5)	509.0(332.5,717.5)	< 0.0001	91.5(24.0,226.5)
Salt submenu**	With	227	417.5(314.0,566.0)	349.0(268.0,476.0)	476.0(362.0,644.0)	< 0.0001	90.0(43.0,170.0)
	Without	52	417.0(285.0,645.0)	376.0(258.0,510.5)	509.0(332.5,717.5)	< 0.0001	91.5(24.0,226.5)
Restaurant category	Chinese cuisine	85	421.0(319.0,653.0)	356.0(289.0,522.0)*	529.0(390.0,715.0)*	< 0.0001	108.0(49.0,223.0)
	Snacks	151	426.0(318.0,545.0)	371.0(277.0,470.0)	476.0(362.0,641.0)	< 0.0001	78.2(37.0,155.0)
	Other	43	350.0(184.0,537.0)	251.0(176.0,509.0)	384.0(312.0,625.0)	< 0.0001	90.0(22.0,193.0)
Dish Category	Meat dish	80	464.5(317.5,621.0)	370.5(286.5,555.0)	544.0(376.5,682.0)	< 0.0001	91.0(41.5,171.0)
	Vegetarian dish	25	371.5(154.0,664.0)	296.0(132.0,529.0)	585.0(317.0,735.0)	0.0209	160.0(47.0,343.0)
	Snack/staple	174	408.0(311.0,517.0)	347.5(268.0,457.0)	464.0(362.0,622.0)	< 0.0001	84.0(37.0,170.0)
Total		279	417.5(310.0,581.0)	352.0(264.0,482.0)	484.0(361.0,653.0)	< 0.0001	90.0(38.0,178.0)

*Compared with the other categories within the Restaurant subgroup, *P* < 0.05

** The restaurants with the salt submenu setup included four intervention groups: Salt submenu with default on “regular salt”, Salt submenu with default on “reduced salt”, Salt submenu with default on “regular salt” + Health message, Salt submenu with default on “reduced salt” + Health message; the restaurants without the salt submenu was equivalent to the group with health message

## Discussion

With the current interface of the leading MDAs in China, if consumers would like to make specific requests related to a dietary preference (e.g., less salt) at the time of the order, the comment box is the primary channel to express such demand. In this study, we found that less than 1% of MDA orders contained consumer comments, meaning that this primary channel to facilitate consumer-specific requests is rarely used. This could be explained by the extra effort required to access and manually type in the comments box, which is often not easily found. Therefore, MDA consumers may be discouraged from requesting their dietary preferences. We observed that when consumers overcame this “extra effort” constraint, a sizeable minority (40.6%) requested less salt. Previous studies in China also have investigated the demand for salt reduction across different populations, and found that approximately 10% of their respondents requested reduced salt food when eating out or ordering take-outs [24, 25]. Together, these findings suggest that consumers in China to some extent have the willingness to make healthier choices, despite the extra effort required to express those preferences when using the current MDAs.

We also surveyed restaurant managers to assess their attitude towards salt reduction, and found that more than 90% of restaurants responding indicated a willingness to use less salt in food preparation if requested by consumers. Furthermore, we verified through our laboratory salinity test that consumers were in fact served reduced salt dishes when they made such requests. Thus, restaurants did transfer their willingness into practice to respond to consumers’ dietary preferences for less salt.

The scale of salt reduction was also measured in our study, with results showing an average reduction of sodium in a dish as 90 mg/100 g. A 2019 study conducted in China reported that the average was 575.6 g per restaurant portion size. A reduced salt version of a dish would contain 518 milligrams less sodium, which is equivalent to a reduction of 1.3 g of salt per dish [23]. In a separate study, statistical modelling found that reducing the Chinese population’s daily salt intake by 1 gram could prevent almost 9 million cardiovascular events by 2030. Thus, reducing salt intake by 1.3 g per dish for meals ordered through MDAs could greatly contribute to public health goals and help to prevent cardiovascular disease in China [26]. It is worth noting that the sodium content of dishes ordered from Chinese cuisine restaurants was significantly higher than that from other types of restaurants, indicating a more notable impact if efforts were made to target and prioritize Chinese cuisine restaurants for salt reduction interventions.

To our knowledge, this was the first study on a MDA in a real-world setting in China to explore consumer demand for salt reduction, and to examine whether consumers’ requests for less salt would result in lower salt content in the deliveries. As described above, although a sizeable minority of consumers had expressed a demand for salt reduction when ordering on the MDA, the constraints posed by limiting consumers to the use of the comment box may have acted as a barrier to expressing a dietary preference, and therefore making healthier meal selections. Thus, it is imperative to create and enable a MDA environment that is conducive to consumers more easily making reduced salt requests and other healthier selections. Policy makers working together with public health authorities and research institutes can take these insights into future work with MDAs to explore accessible pathways for salt reduction on digital platforms, as well as measure the population impact of this future work. One potential pathway is the wider use of the salt submenu, which was featured and tested for effectiveness in our whole project, and presented in another manuscript. Additional initiatives should extend the findings of other studies aiming to support healthier food choices and behavior changes [27–29]. Importantly the MDAs and participating restaurants also carry the responsibility to support consumers in making healthier meal choices. Through a joint effort, we can shape the food environment and make salt reduction an easier option for consumers [30].

Limitations to the study include that as part of the whole project on salt reduction, the surveyed and sampled restaurants may already have a high willingness to promote salt reduction and provide reduced salt dishes, as compared with those that did not sign up for the study, which can contribute to bias in this study.

## Conclusions

Many Chinese consumers request less salt in their ordered meals; however, the current setup on MDAs may make it difficult for consumers to make this request. Restaurants demonstrated a strong willingness to respond to consumers’ specific dietary requests, and prepare meals for delivery with less salt if requested by consumers. Though further research is needed to cover restaurants in a broader demographics, it is clear that a more enabling food environment on MDAs is a key element to support healthier choices for meals prepared outside the home. But it requires multisectoral collaboration among policy makers, researchers, consumers and the MDAs. With collective efforts, we can make reduced salt options more accessible to and effortless for consumers, and contribute to the health benefits across the population.

## Data Availability

The data of consumer comments on the MDA are not publicly available, but the data of survey and sodium content are available from the corresponding author on reasonable requests.

## Abbreviations

CVDs: Cardiovascular diseases
WHO: World Health Organization
MDAs: Meal delivery apps
