# Correction: Do restaurants comply with reduced salt requests from consumers ordering on meal delivery apps?

**DOI:** 10.1186/s12889-023-17453-2

**Published:** 2024-01-03

**Authors:** Chao Song, Wenyue Li, Ying Cui, Beisi Li, Zhongdan Chen, Paige Snider, Ying Long, Ailing Liu, Gauden Galea

**Affiliations:** 1 National Institute for Nutrition and Health, Chinese Center for Disease Control and Prevention, 100050 Beijing, China; 2 School of Architecture, Tsinghua University, Beijing, China; 3 World Health Organization Representative Office in China, Beijing, China

**BMC Public Health (2023) 23:2000**

10.1186/s12889-023-16939-3

The original publication of this article contained an incorrect license and copyright holder. The incorrect and correct information is shown in this correction article. The original article has been updated.

Incorrect

© The Author(s) 2023. Open Access This article is licensed under a Creative Commons Attribution 4.0 International License, which permits use, sharing, adaptation, distribution and reproduction in any medium or format, as long as you give appropriate credit to the original author(s) and the source, provide a link to the Creative Commons licence, and indicate if changes were made. The images or other third party material in this article are included in the article’s Creative Commons licence, unless indicated otherwise in a credit line to the material. If material is not included in the article’s Creative Commons licence and your intended use is not permitted by statutory regulation or exceeds the permitted use, you will need to obtain permission directly from the copyright holder. To view a copy of this licence, visit http://creativecommons.org/licenses/by/4.0/. The Creative Commons Public Domain Dedication waiver (http://creativecommons.org/publicdomain/zero/1.0/) applies to the data made available in this article, unless otherwise stated in a credit line to the data.

Correct

© World Health Organization 2023. Open Access This is an open access article distributed under the terms of the Creative Commons Attribution IGO License (http://creativecommons.org/licenses/by/3.0/igo/legalcode), which permits unrestricted use, distribution and reproduction in any medium, provided you give appropriate credit to the original author(s) and the source, provide a link to the Creative Commons license, and indicate if changes were made. In any reproduction of this article there should not be any suggestion that WHO or this article endorse any specific Organization or products. The use of the WHO logo is not permitted. This notice should be preserved along with the article’s original URL.

